# Cooperation and Defection at the Crossroads

**DOI:** 10.1371/journal.pone.0061876

**Published:** 2013-04-16

**Authors:** Guillermo Abramson, Viktoriya Semeshenko, José Roberto Iglesias

**Affiliations:** 1 Centro Atómico Bariloche, CONICET and Instituto Balseiro, Bariloche, Argentina; 2 Facultad de Ciencias Económicas, Universidad de Buenos Aires, Buenos Aires, Argentina; 3 Instituto de Fsica, UFRGS, Programa de Pós-Graduação em Economia, UFRGS, and Instituto Nacional de Ciência e Tecnologia de Sistemas Complexos, Porto Alegre, Brazil; University of Maribor, Slovenia

## Abstract

We study a simple traffic model with a non-signalized road intersection. In this model the car arriving from the right has precedence. The vehicle dynamics far from the crossing are governed by the rules introduced by Nagel and Paczuski, which define how drivers behave when braking or accelerating. We measure the average velocity of the ensemble of cars and its flow as a function of the density of cars on the roadway. An additional set of rules is defined to describe the dynamics at the intersection assuming a fraction of drivers that do not obey the rule of precedence. This problem is treated within a game-theory framework, where the drivers that obey the rule are cooperators and those who ignore it are defectors. We study the consequences of these behaviors as a function of the fraction of cooperators and defectors. The results show that cooperation is the best strategy because it maximizes the flow of vehicles and minimizes the number of accidents. A rather paradoxical effect is observed: for any percentage of defectors the number of accidents is larger when the density of cars is low because of the higher average velocity.

## Introduction

Urban transportation systems are a source of numerous inefficiencies and of negative externalities. Traffic problems worsen due to heavy congestions; additionally there are environmental issues such as smog and noise pollution, and huge economic losses due to congested traffic. In order to improve efficiency and reduce externalities it is important to understand traffic dynamics in a controlled environment and to identify optimal control strategies which could help alleviate the problem.

Traffic flow problems have received much attention for decades. Many investigations have been carried out using different points of view and considering various aspects of traffic phenomena, and are evaluated in order to better understand the overall quality of traffic flow. One of the main questions in the study of traffic is how to better accommodate the demand for mobility in a system.

The pioneering traffic flow descriptions on a highway are derived from observations made by Greenshields, first published about 75 years ago [2]. Greenshields carried out tests to measure traffic flow, traffic density and velocity using photographic measurement methods for the first time. He was able to develop a model of uninterrupted traffic flow that predicts and explains the trends which are observed in real traffic.

Nowadays the search for the mechanisms behind the complex interactions between drivers, vehicles and road infrastructure continues. Also, traffic congestion has deteriorated considerably. Recently traffic problems have attracted the attention of physicists because of observed non-equilibrium properties and various nonlinear dynamics phenomena. Several approaches have been proposed to investigate the behavior of vehicular traffic. Most of the approaches are classified into macroscopic and microscopic models based on how the movement of vehicles is considered.

In the macroscopic approach a traffic stream is viewed as a continuous medium. The collective vehicle dynamics is described in terms of the spatial vehicular density per lane and the average velocity as a function of the freeway location and time. The first major step in macroscopic modeling of traffic was carried out by Lighthill and Whitham in 1955 [3], when they compared the “traffic flow on long crowded roads” with “flood movements in long rivers”. A year later, Richards (1956) [4] complemented the idea by introducing “shock-waves on the highway” with an identical approach. That is the origin of the LWR model. It is common to refer to this class of models as first-order models. Another kind of macroscopic model, second-order models, contain an additional partial differential equation for the average velocity and take into account the finite relaxation time to adapt the velocity to changing traffic conditions [5], [6].

In the microscopic approach the motion of each vehicle in a traffic stream is considered. Thus, the dynamic variables of the model represent microscopic properties such as the position and velocity of a single vehicle. The so-called car-following models focus on the non-linear interaction and dynamics of single vehicles. The driving behavior of a vehicle depends significantly on the motion of the preceding vehicle: the acceleration is a function of the vehicle’s distance to the preceding one and of its own and relative velocities [7]–[9]. These models are used only for detailed studies (e.g. on-ramp traffic, bottlenecks, effects of traffic optimization measures), as they consume an enormous amount of CPU time because of the large number of variables involved. An alternative approach are cellular automaton (CA) models, which permit the simulation of a minimal model of traffic dynamics faster than real-time simulations [1], [10], [11]. Cellular automata use integer variables to describe the dynamic properties of the system by discretizing space and time. The Nagel-Schreckenberg (NaSch) model [10] is a basic CA model describing a one-lane traffic flow. Based on this model many CA have been extended to investigate the properties of systems with realistic traffic factors such as highway junctions, crossings, tollbooths and speed limit zones [12]–[14].

An extensive and generous overview of traffic modeling can be found in a review article by Helbing [15]. He considers empirical data and reviews the main approaches to modeling pedestrian and vehicular traffic. Control strategies including ramp metering [16], [17], and variable speed limits [18] have also been widely studied.

At the most basic level, traffic dynamics are often discussed on a homogeneous roadway. Next it becomes necessary to consider road intersections. Modeling intersections is difficult, since intersection models are phenomenological by nature. They describe, for instance in the case of a merge, the local priority rules.

At an intersection a limited space must be shared by vehicles from different directions. Various approaches have been used to resolve the obvious traffic conflicts. There are schemes that require a vehicle to come to a full stop, e.g. stop signs or traffic lights. Other types of schemes try to avoid the full stop of vehicles, like traffic circles or roundabouts [19], [20].

In this paper we extend the original discrete model proposed by Nagel and Paczuski [1] in order to account for a non-signalized intersection. This is a common problem in street intersections within cities, particularly in old cities where crossings are neither rotatory nor signalized. Earlier, Zhang *et al.* [21] considered the intersection problem within a game-theory framework, and will be revisited in the next sections. Perc [22] has also studied the effect of competing strategies in a different discrete traffic model. In this paper we study the effects of cooperator or defector behavior on the flow and average velocity of vehicles, as well as the incidence of accidents when cars do not stop at crossings.

The paper is organized as follows. In the next section we define the model and the rules that describe the behavior of vehicles on the street and at an intersection. Next we describe the setting of the simulations and present the results obtained. Finally, we conclude and discuss the potential relevance of this work to the solution of the problems of real traffic.

## Model and Methods

Let us present a model for traffic dynamics at a single intersection and describe the flow in the system. We choose to model the motion of a vehicle on a single lane street using the automaton rules proposed by Nagel and Paczuski [1]. The interaction of vehicles arriving at the intersection, and which one is going to pass first, is determined by the set of rules that we will specify hereafter.

**Figure 1:**
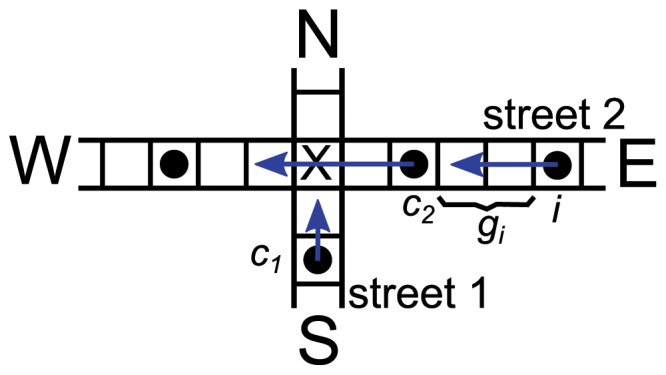
Two intersecting streets. Cartoon showing the geometry of the model and basic notation.

We consider a system of two streets, 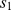 and 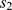, that cross at a given point 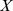. The streets have a defined sense of circulation: South to North for 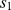 and East to West for 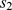 (see Fig. 1). Each street is a ribbon with 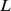 slots, with periodic boundary conditions. On each street we place 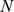 vehicles (initially at random), giving linear density 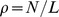, and identified by an index 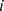. Double occupancy of the sites is prohibited (except at 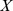). Following Ref. [1] we consider a variable associated with the distribution of cars in the streets, the gap 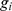, that is the number of empty sites in front of car 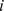 up to the car ahead. At the intersection the gap needs further specification, as will be explained below.

Each car has a time-dependent velocity 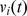 that takes discrete values between 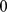 and 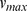. Time proceeds discretely, and 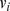 is the number of sites each vehicle advances during one time step. At the beginning of the simulation all cars have zero velocity.

For the purpose of defining the interaction at the crossroad we identify the cars nearest to the intersection as 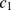 and 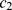 (in streets 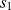 and 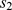 respectively). Streets are equivalent, i. e. the same traffic rules are applied to both streets.

### General rules of vehicle motion

Firstly, let us describe the dynamics of a vehicle away from the intersection. For every configuration of the model, one iteration consists of the following steps, performed simultaneously for all vehicles:

1. If 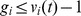, car 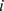 will reduce its velocity as follows:
  - With probability 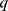: 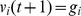.
  - With probability 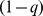: 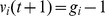 (overbrake, with a further reduction of velocity).
2. If 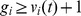 and 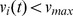, the car will accelerate as follows:
  - With probability 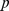: 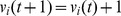.
  - With probability 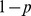: 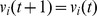 (keep the same velocity).
3. If 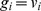, or if 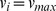 and the gap is 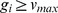, the velocity 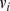 does not change.
4. After updating the velocities, each car advances 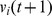 sites.

Note that, in rule 2, Nagel and Paczuski [1] consider 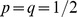. We prefer to keep some flexibility in the choice of the probabilities. In the simulations reported below we set 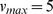, but any value 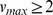 gives the same qualitative behavior.

### Dynamics at the intersection

In order to define the rules that regulate the movement of vehicles at the uncontrolled intersection, one needs to determine the priorities when crossing the intersection, just like in real crossroads, making the transit fluid and avoiding collisions.

Zhang *et al.* [21] considered a similar problem in a game theoretical framework. In their model, drivers approaching the intersection behave either as cooperators (C) or defectors (D), allowing (or not) the cars arriving on the other street to pass. Furthermore, drivers always adopt complementary strategies, that is, if 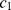 is C (D), then 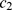 is D (C). Thus the dynamics at the intersection is deterministic, since all pairs are of C–D or D–C type, and cooperators will always let the defectors cross. Zhang *et al.* set the probability of cooperating between 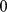 and 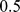 (and the probability of defecting between 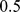 and 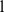). Since cooperators stop at the intersection, the street with the larger number of defectors exhibits an average velocity higher than the other one. However, the average total flow of both streets appears to be rather independent of the probability of cooperation.

We believe that the use of cooperating and defecting strategies is a fair approach for the description of the interaction of drivers at crossroads in many real situations. A true game description must take into account the full set of possible interacting strategies. That is, drivers arriving at the intersection may as well be both cooperators (C–C) or defectors (D–D). Since the authors of Ref. [21] penalize only cooperators, it is better to be a defector, and it results that a driver should not behave *a priori* in a cooperative way. It is precisely due to the relative payoffs of the complete set of interactions that the formal games of Hawks and Doves or Prisoner Dilemma gain their interest in the description of social systems.

In our model a driver has a strategy that determines his behavior at the intersection. These are set at random at the beginning of the simulation, with probability 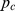 for cooperation and 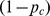 for defection. In order to avoid deterministic or synchronization artifacts arising from the periodic boundary conditions, when a car reaches the end of the lane and re-enters the street, drivers are reassigned new strategies, at random with the same probability 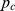. In this way, heterogeneity in drivers’ behavior is incorporated in the model.

Let us specify the interaction at the crossroad in a way that imitates what happens in real situations. We impose a single traffic rule:

- Rule 1: Drivers must always yield to cars approaching from the right.

Rule 1 is a widespread right-of-way traffic rule that applies for equivalent streets in the absence of control devices in almost all countries with a right-hand driving. If street 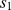 runs from South to North, and street 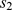 from East to West (see Fig. 1), the driver 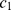 must respect the priority of 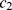 and let him pass first. However, traffic rules are not always respected, and some drivers may try to cross disregarding the rule. This behavior may impact the traffic flow in different ways depending on the density of cars, and it is the phenomenon that we aim to study.

We define the following strategies:

- Cooperate: abide by Rule 1.
- Defect: ignore Rule 1.

The result of the interaction can be quantified in terms of a payoff (rather, a *cost*) as the time that it takes to cross the intersection. Cooperation results in a fluid flow, while interactions D–C, C–D or D–D produce delays in the crossing. In order to implement this we need to set up rules to define the gaps and velocities of cars 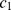 and 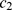, in addition to those of the rest of the system. These are the following:

1. Determination of the gaps:
  - If 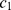 (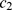) is cooperator: The gap is measured as the distance from the car to the intersection 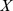 (cooperators slow down when approaching the intersection). (This rule applies only if the car is at a site strictly less than 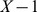, otherwise it would give 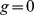 and it would stop.).
  - If 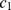 (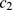) is defector: measure the gap as usual, up to the car ahead.
  - For either case: if there is a car at the intersection *driving from the other street*, measure the gap up to 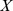 (neither C or D will crash intentionally).
2. Determination of velocities:
  - If 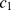 is cooperator and is at site 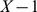, it yields. If the velocity of 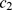 is such that it will cross the intersection, 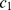 sets its velocity to 0 (“stop at the intersection”). However, if the speed of 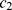 is not large enough to cross at that step, 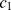 sets its velocity according to the general rule and keeps advancing. Note that 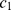 yields disregarding the strategy of 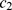.
  - If 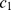 is defector and 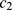 is cooperator, both may try to cross at the same time (if their velocities are large enough to allow it in the current time step). In this case, the velocities are set up in such a way that cars advance only up to the intersection (not further, not before). This rule slightly favors the right hand driver (the cooperator) with respect to the left hand one (defector), by penalizing the defector (with a reduction of speed). However, neither car stops. This simulates an “almost crash”, where both drivers lose some time (the defector more than the cooperator). At the next time step they will accelerate if it is allowed by the traffic density.
  - If 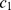 and 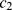 are defectors, both may try to cross at the same time (again: both speeds need to be large enough given their current positions). In this case neither car yields, and we penalize both of them with a crash. Cars are given velocities just enough to bring them to the intersection, and they are flagged to have their speeds set to 0 *the next time step*. So they will stay at 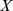 one more time step, causing interruption of the traffic flow, which will pile up behind them. The next time step cars will accelerate if allowed by the traffic density.

Given this set of rules, we performed various simulations. In the next section, we present the results obtained.

## Results and Discussion

In the simulations, the length of each street is 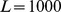 sites. 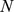 cars are distributed at random in each street, giving a density 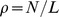. Starting with an initial condition in which all vehicles have zero velocities, we wait a reasonable transient time in order to obtain a stationary phase, defined by the average velocity in both streets. We then calculate the flow of cars, defined as the average velocity times the density, 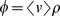, additionally averaged over the stationary state. We also calculate various statistical properties of the distribution of velocities.

**Figure 2:**
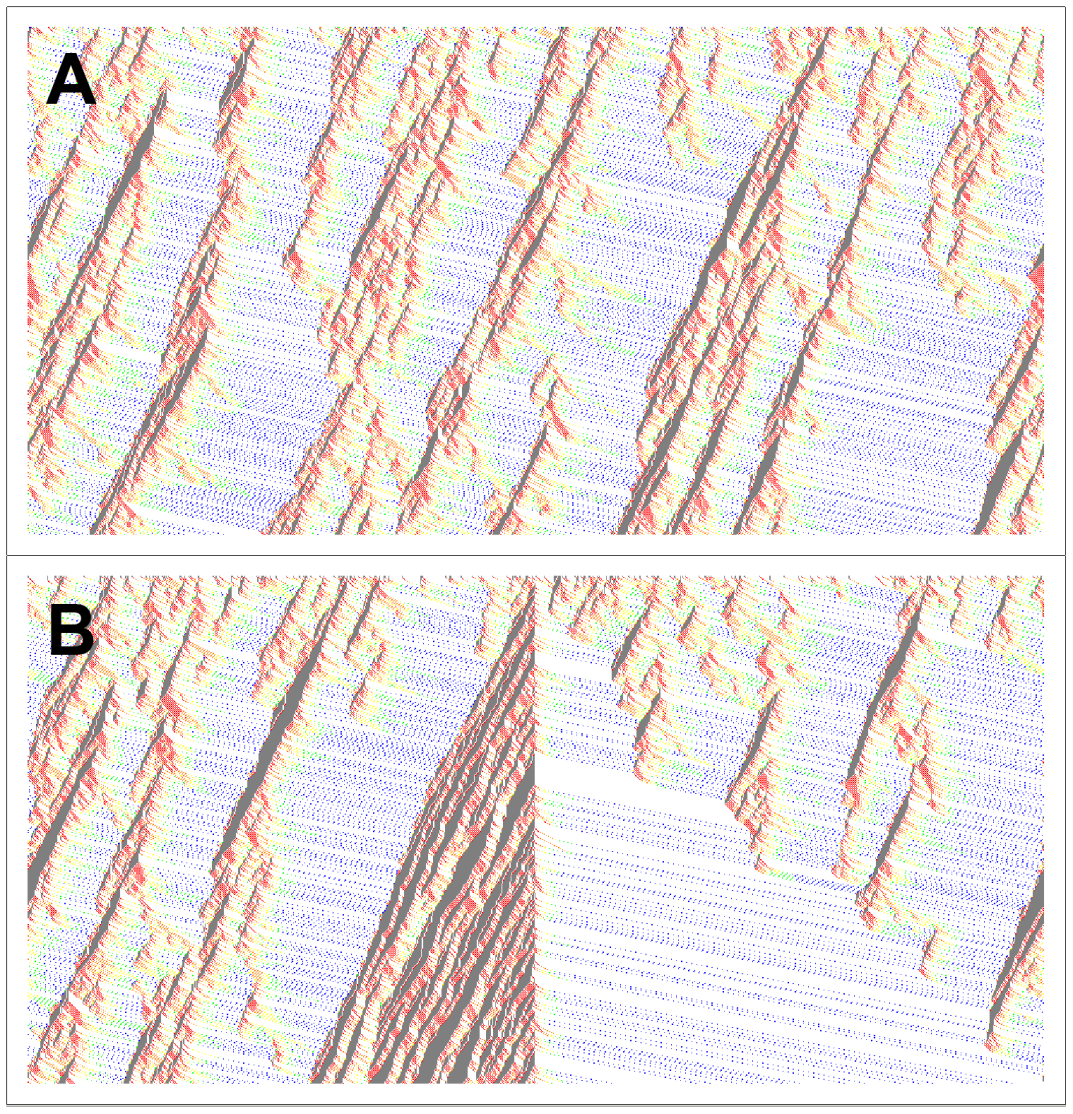
The ordering effect of the intersection. Plot of the car’s positions (horizontal axis) as a function of time (vertical axis) for 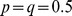, 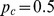, 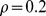. Cars move from left to right and the time increases downwards. A: Nagel-Paczuski model [1]; B: our model (street 1). Empty sites are represented by *white* dots, sites that are occupied by a car are represented by a specific colored dot, where different colors correspond to different velocities. *Red* dots stand for the cars with velocity 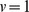, *orange* for 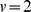, *yellow* for 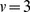, *green* for 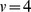, *blue* for 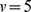, and *black* for 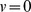. Note the backward motion of the traffic jams.

Let us first observe a comparison between a pure Nagel system and street 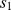 of our model (Fig. 2). System parameters correspond to a rather noisy behavior of the drivers, with 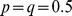, and a density of 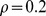 cars per site. For the case of the pure Nagel system (see Fig. 2.A), there are congestion clusters (jams), which are formed randomly due to velocity fluctuations of the cars. These cars either stop moving or move very slowly, and can accelerate to full speed only after having left the jam, keeping this velocity until the next one. Thus, the stationary state is characterized by an inhomogeneous mixture of jam free regions and higher density jammed regions. These jammed regions decrease the average flow in the system. In Fig. 2.B we show the results of our model. We observe that, from the beginning, the intersection acts as an ordering defect. Even if its action is local, its effect is far reaching. When the cars reduce their velocity, and even stop at the intersection, a free space is created ahead of the defect. After crossing the intersection cars can accelerate to maximum velocity, resulting in an almost completely ordered flow that persits downstream. So, the intersection acts as a source of order in the traffic. By allowing vehicles to pass one at a time it effectively destroys the spontaneous jams observed by Nagel.

**Figure 3:**
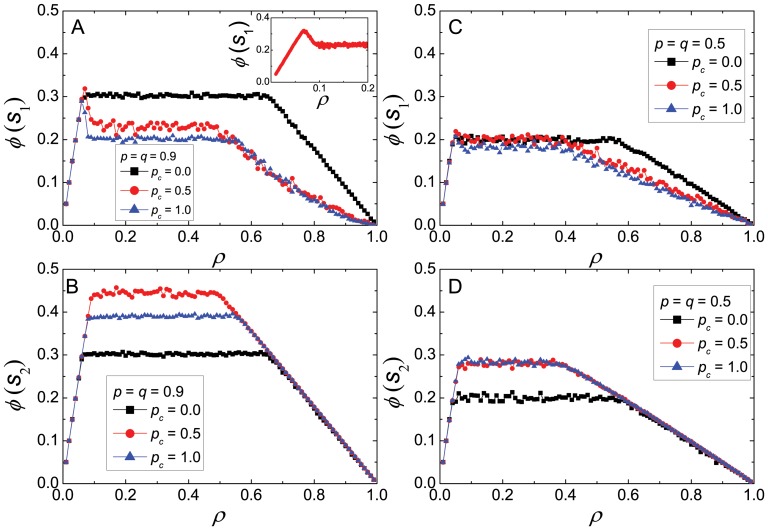
The three phases of traffic. The flow as a function of the density in streets 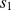 [A and C] and 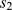 [B and D]. A system with 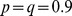 appears in the left column [A and B], with the more noisy case of 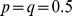 shown next to it [C and D]. The curves show the behavior for three different values of the probability of cooperation 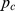: 0, 0.5 and 1, as shown in the legends.

Now we study macroscopic fundamental flow diagrams for a variety of traffic scenarios. These diagrams show the relation between the flow and the density and are represented in Fig. 3. Three phases are observed: (1) a low density phase, with freely flowing traffic at the maximum speed (where the flow grows linearly with the density); (2) a high density phase, corresponding to heavily congested traffic and very slow speed, with the flow depending inversely on the density of cars; (3) an intermediate density phase where the flow remains in *a plateau independent of the density* and thus the average velocity is in inverse proportion to the density. The first two phases are also present in Nagel-Paczuski’s model [1]; the third phase has been observed by Zhang *et al.* [21]. The transition between the free flow phase and the plateau is a crossover that, in street 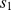 (panels A and C in Fig. 3), appears as a peak. We looked at the dynamics in this region in detail, and this peak does not correspond to any abrupt phase transition. A close up of one of the peaks appears as an inset in Fig. 3.A. The fast reduction of flow in the yielding street (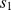) must be interpreted, precisely, as the yielding vehicles stopping, at the first stages of the jamming produced by an increased density. We remark that, since the flows in both streets are considered separately, the intersection can be seen as a defect in the street. However, the fact that one of the streets is the preferential one makes the flow different in the yielding street and the preferential one.

We performed simulations for two pairs of values of the probabilities 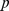 and 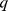 (see Model and Methods). On one side, 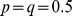 represents the behavior of undecided or cautious drivers, which we call a noisy system. These are drivers that half of the time do not accelerate to the maximum possible velocity, and the rest of the time brake more than it is strictly necessary. This set of values has been used by Nagel and Paczuski [1], and will serve as a reference. Another pair of values, 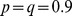, represents more “deterministic” drivers, who mostly try to optimize their motion. Observe, in Fig. 3, that the behavior of the system is qualitatively the same in both cases. Nevertheless, the flow of the noisy system is a little slower than the more deterministic one (the corresponding curves on the left panels of Fig. 3 are higher than those on the right). In addition, we explored a wide range of values for the probability of cooperation 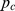, ranging from zero cooperators to a fully cooperating system. As expected, the flow is the same in both streets when 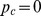 (all defectors, black squares). When 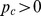 the flow is greater in street 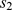 than in street 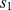. Observe that the impact of cooperation is less relevant in the noisy case, 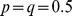.

**Figure 4:**
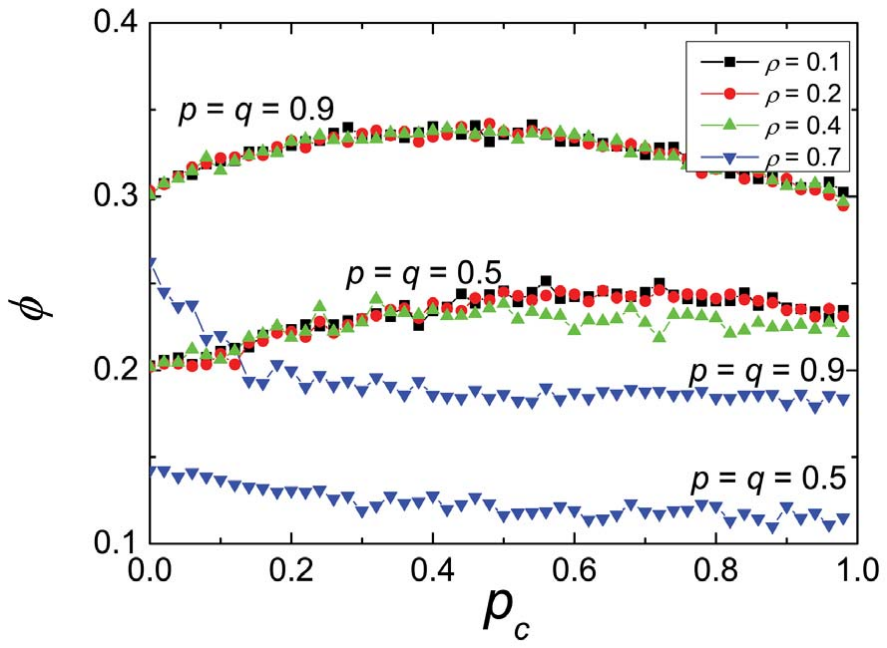
The effect of cooperation. The total flow 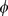 vs. the probability of cooperation 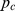, for different values of the density. For small and medium densities the flow is not monotonous, showing a maximum at an intermediate value of 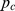. For high densities the flow decreases with 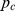.

On Fig. 4 we plot the *total* flow as a function of the probability of cooperation 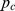, for several fixed values of the density, in order to visualize the effect of cooperation. This is shown for the two scenarios, 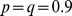 and 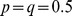. The trends are similar in both cases, even though the flow is greater for the deterministic case. More interesting than this is the fact that, for less congested systems, the dependence on cooperation is non monotonous. There is a *maximum* flow at an intermediate value of 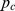, indicating that an excess of cooperation may induce a jam at the intersection. On the other hand, for high densities (e.g. 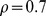, as shown) the flow monotonically decreases with the probability of cooperation. Indeed, scenarios with very high densities usually perform better, in real life, when drivers switch their strategies to a new one (neither C nor D), alternating turns to cross the intersection, instead of stopping constantly yielding to the vehicles on the right.

**Figure 5:**
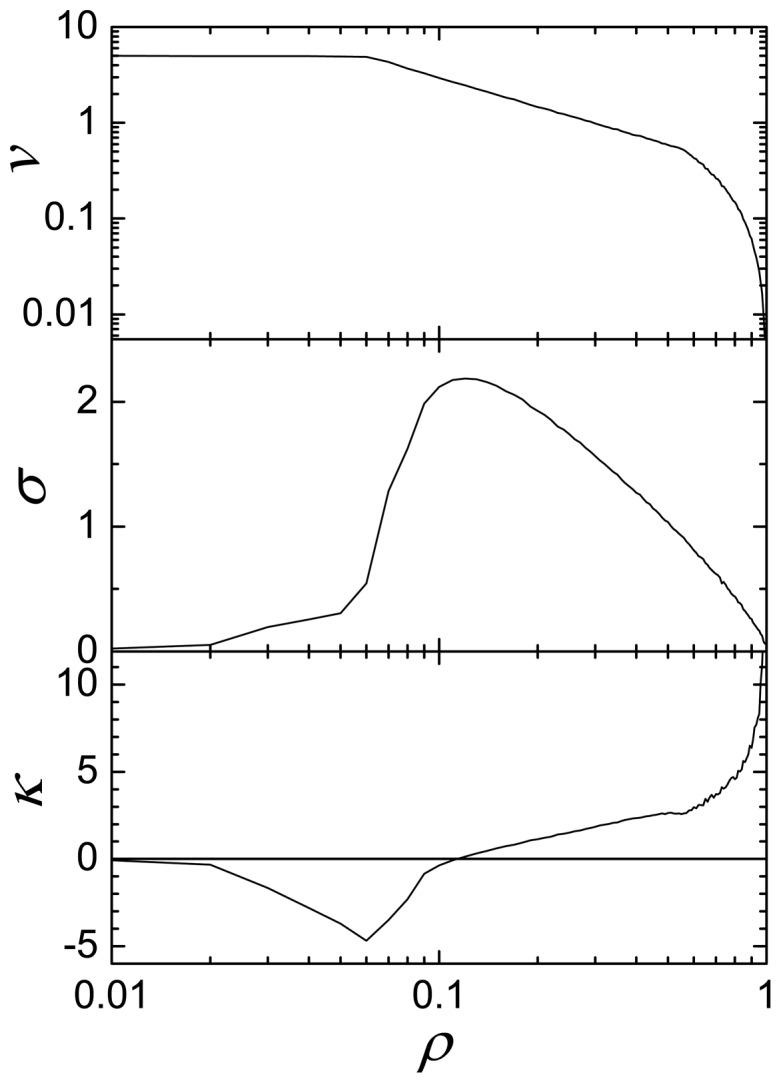
The distribution of velocity as a function of density. The average velocity , the standard deviation and the skewness of the distribution are plotted for 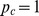 and 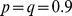.

We must remark that the flow is an average measure of the traffic. In order to get a complementary description we analyzed the distribution of velocities. On Fig. 5 we plot the mean value, 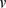, the standard deviation, 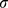 and the skewness, 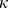 of this distribution, as a function of the density 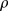. One can see that for small densities the velocity stays very near the maximum 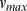 (laminar flow), and then sharply decreases when the cars start to pile up at the intersection jam. The intermediate density region is a perfect inverse power law 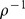, corresponding to the plateau of the flow that we showed before. A break in this law is seen at 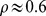, corresponding to the beginning of the high density regime. The standard deviation is close to zero in the laminar flow region. Then, it starts to grow when the flow enters in the plateau region and exhibits a maximum for intermediates values of the density, indicating a big dispersion in the velocities. The dispersion diminishes for high values of the density: the traffic becomes more uniform and slower. For very high densities the average velocity is very small and so is the standard deviation. The skewness of the distribution complements this information. For small densities it is negative, since the maximum of the distribution corresponds to large values of the velocity. When increasing the density the skewness goes through zero, indicating a symmetric distribution. For high values of the density the distribution is centered at low velocities, and the skewness is positive.

**Figure 6:**
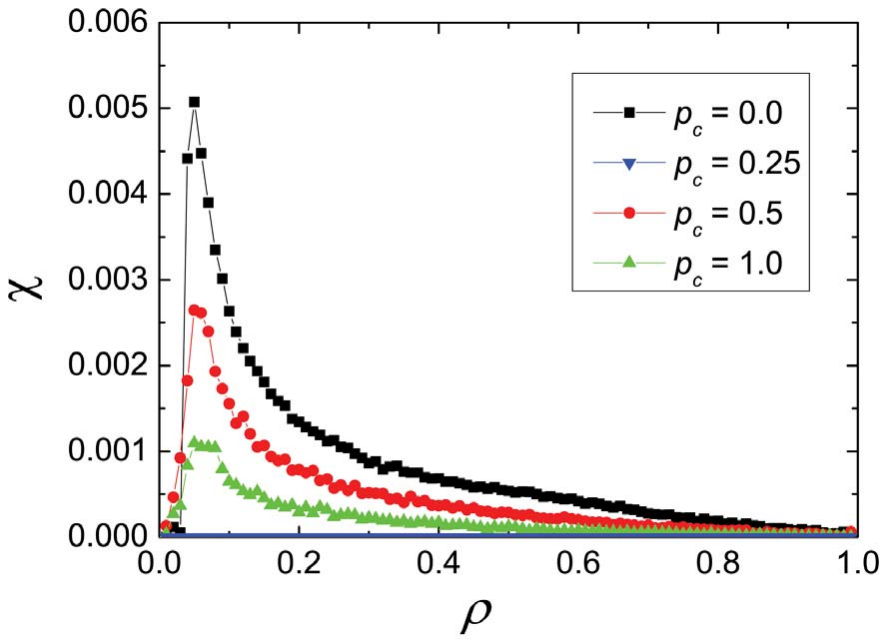
Crashes. Number of crashes per cars per unit time, as a function of density. The curves correspond to the case 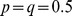, for three values of the density of cooperators (as shown in the legend).

Another important variable to consider is the number of crashes. A crash does not only cause a time delay in traffic but also, in real systems, has an economic impact. In Fig. 6 we plot the number of crashes per car and per unit time, 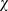, as a function of the density of traffic, for different values of the probability of cooperation 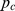. One can see that there is a peak in the number of accidents for low densities. This is due to the fact that even though the number of cars is small they move fast, and then the number of accidents per unit time is large. On the contrary, when the density is high the average velocity is small and the number of accidents per unit time decreases almost to zero. For all densities, we also verify that the number of accidents decreases when increasing the probability of cooperation (and goes to zero when all drivers are cooperators). Also, more deterministic systems (not shown), display more crashes due to the higher vehicle speed.

**Figure 7:**
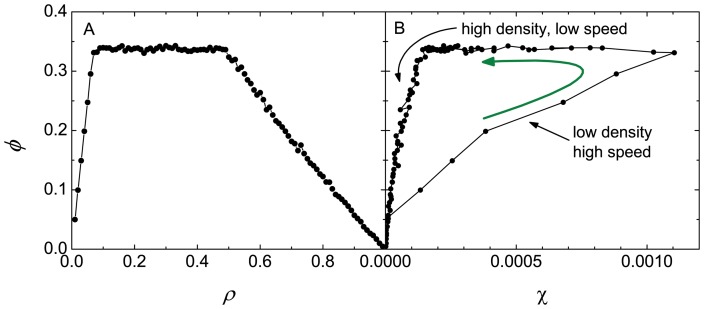
Correlation between crashes and flow. Relation between the flow and the number of crashes per car and per unit time for 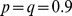 and two values of 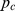. In the right panel (B), follow the direction of the arrow when reading the description in the text.

The correlation between the rate of crashes and the flow is analyzed in Fig. 7. An hysteresis loop is observed. When the flow increases at a low density the number of crashes increases very fast (due to high vehicle speed), attaining a maximum when the flow enters the plateau (for a density 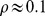). Within the plateau the number of crashes diminishes and when the flow decreases at high densities the number of crashes further decreases going finally to zero at very high densities where the traffic goes to a standstill.

**Figure 8:**
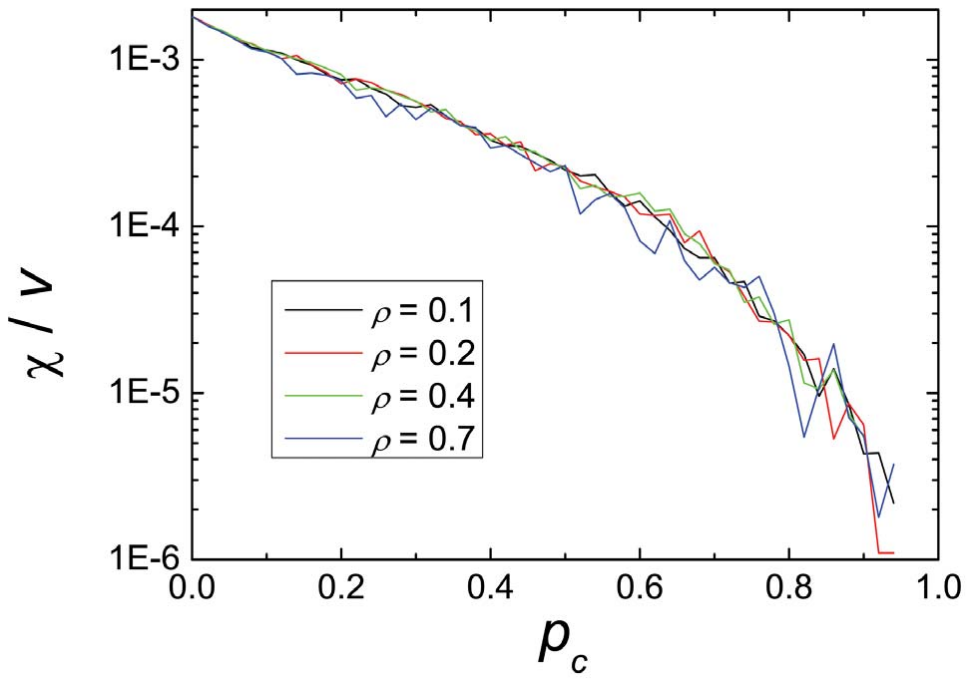
Universality of crashes and cooperation. Crashes vs 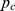 in the case 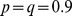, shown for four values of vehicle density.

Finally, in Fig. 8 we show the number of crashes normalized by the average velocity, 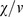, vs the probability of cooperation 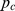. This function decreases in a monotonic way when increasing the probability of cooperation, as expected. Moreover, as the curves corresponding to four values of vehicle density show, there is a nearly universal exponential behavior as a function of the cooperation, which is independent of the density. This behavior could provide a good field test of our model in real situations. This will be studied in further work.

### Alternative rules

The rules of interaction between a defector in 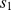 and a cooperator in 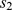 (rule 2.b) imply some advantage of the latter, which is the opposite of some paradigmatic games of defection and cooperation like the Prisoners’ Dilemma. In order to study this point we have analyzed some alternative rules to 2.b. They may be summarized as follows:

**2.b1)** The defector keeps his velocity as if the intersection did not exist, while the cooperator reduces his velocity to arrive just to the intersection at 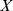. This rule clearly favors the defector by penalizing the cooperator (who has the priority). The cooperator is obliged to reduce his velocity and the situation is an almost crash, but differently from the original rule, the defector is not penalized. At the next time step the cooperator 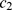 accelerates from the point 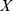.

**2.b2)** A slight variant of 2.b1: 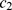 reduces his velocity to arrive just to the intersection 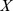 and there *stops*, i.e. it will continue in the next step accelerating from 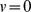. The situation for the defector is the same as in rule 2.b1.

It is interesting and reassuring that these alternative rules produce no significative changes in the presented results and for this reason we do not include new figures. Some minor differences arise when considering rule 2.b2 because the cooperators driving in street 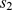 are obliged to stop completely at the intersection. Thus, they exhibit a lower average speed. But these changes are not relevant enough and show only small variations in the numerical values of the measured variables when compared with the general results already presented.

## Conclusions

We studied the flow and speed of cars circulating on intersecting one-lane streets, and where drivers coming from the right have the precedence. The drivers may be cooperators—when they respect the right precedence—or defectors if they ignore this rule. We observed some significative trends in the results, that we detail below.

The flow increases linearly with the density of cars for very low densities and then remains constant for a wide range of densities. For high densities the flow decreases dramatically to values very near zero (see Fig. 2). This indicates the existence of two critical densities: the first one when the system enters into the plateau of constant flow and the second when it leaves the plateau. Within the plateau region the flow is constant, suggesting that the street has a “capacity” up to 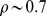. However, the width of the plateau decreases with the number of defectors both in streets 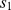 and 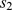. Also, “undecided” drivers that accelerate less that the maximum possibility or brake more than needed reduce the global performance: the flow is much lower when 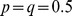 than when 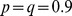.

These results can be confirmed by observing the velocity as a function of the density (Fig. 5). The velocity is maximum for low densities, then decreases in inverse proportion to the density for intermediate values and reaches zero for high densities. Again, the plateau region provides a good traffic flow, but the dispersion of the speeds is high.

It is curious that the behavior of cooperation or defection is not very relevant. Indeed for intermediate or high densities of cars it is more important to be a “decided” driver, accelerating or breaking the maximum or minimum respectively, than to cooperate. The maximum flow is obtained for half of the drivers being defectors and for intermediate densities, or when all the drivers are defectors for high densities. As a matter of fact, for high densities the absolute respect of the right hand precedence can completely block the circulation in street 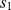, a well known phenomenon in many real traffic situations.

Nevertheless one must keep in mind that the defectors may be particularly dangerous when the density is low. In this case the average speed is high and the number of accidents can also be very high (see Fig. 7).

One can conclude that a significant fraction of defectors is very dangerous at low densities, or in regions of high speed, but a number of them is necessary to increase the flow at intermediate or high densities of cars. Indeed for very high densities the righ hand precedence is annoying and alternate crossing should be preferred.

Comparison with real data are in progress, as is also the study of two-lane streets and the comparison between non-signaled and signaled crossings. Also, we plan to extend our model to the study of two-dimensional block model cities, in the line of Refs. [23], [24].
